# The limited prognostic role of echocardiograms in short-term follow-up after acute decompensated heart failure: An analysis of the Korean Heart Failure (KorHF) Registry

**DOI:** 10.1371/journal.pone.0188938

**Published:** 2017-12-19

**Authors:** Sung Hea Kim, Hyun-Joong Kim, Seongwoo Han, Byung-Su Yoo, Dong-Ju Choi, Jae-Joong Kim, Eun-Seok Jeon, Myeong-Chan Cho, Shung Chull Chae, Kyu-Hyung Ryu

**Affiliations:** 1 Department of Cardiology, Konkuk University School of Medicine, Dongtan Sacred Heart Hospital, Hwaseong, Korea; 2 Department of Cardiology, Hallym University College of Medicine, Dongtan Sacred Heart Hospital, Hwaseong, Korea; 3 Department of Internal Medicine, Yonsei University Wonju Christian Hospital, Wonju, Korea; 4 Department of Internal Medicine, Seoul National University College of Medicine, Bundang Hospital, Seongnam, Korea; 5 Department of Internal Medicine, University of Ulsan College of Medicine, Asan Medical Center, Seoul, Korea; 6 Department of Internal Medicine, Sungkyunkwan University College of Medicine, Samsung Medical Center, Seoul, Korea; 7 Cardiology Department, Chungbuk National University College of Medicine, Cheongju, Korea; 8 Department of Internal Medicine, Kyungpook National University College of Medicine, Daegu, Korea; Universita degli Studi di Roma La Sapienza, ITALY

## Abstract

**Background:**

The prognostic values of the left ventricular ejection fraction (LVEF) and end-diastolic dimension (LVEDD) have primarily been shown among patients with chronic heart failure (HF), with little representation of patients with acute HF (AHF). Therefore, we investigated the value of these echocardiographic parameters in predicting clinical outcomes among patients in the Korean Heart Failure (KorHF) Registry.

**Methods:**

The KorHF Registry consists of 3,200 patients who were hospitalized with AHF from 2005 to 2009. The Kaplan-Meier method was used to estimate survival and readmission, and differences were assessed using the log-rank test. Predictors of survival were identified using univariate and multivariate Cox proportional hazards regression analyses.

**Results:**

Echocardiograms from 2,910 of the 3,200 patients (90.9%) were evaluated. The median LVEF and LVEDD (37% and 56 mm, respectively) were used as cut-offs for the binary transformation of each parameter. The cumulative death-free survival rates for all patients did not significantly differ based on LVEF or LVEDD quartiles; however, an LVEF greater than the median was associated with a better prognosis in ischemic HF patients (log-rank test; p = 0.039). Among ischemic HF patients, LVEF (dichotomized) was a significant predictor of death in a Cox model after adjusting for a history of HF, age, systolic blood pressure (SBP), serum sodium, sex, diabetes mellitus (DM), chronic obstructive pulmonary disease (COPD), chronic kidney disease (CKD), acute myocardial infarction (AMI), atrial fibrillation (Af) and anemia (hazard ratio (HR) 1.475, 95% confidence interval (CI) 1.099–1.979, p = 0.010). The cumulative readmission-free survival rates significantly differed among ischemic HF patients only when based on LVEDD quartiles (log-rank test; p = 0.001). In multivariate Cox proportional hazards regression analyses, LVEDD (dichotomized) remained a significant variable only among patients with ischemic HF after adjusting for sex, age, AMI, DM, COPD, serum sodium, SBP, blood urea nitrogen (BUN) and anemia (HR 1.401, 95% CI 1.067–1.841, p = 0.015).

**Conclusions:**

Among ischemic AHF patients in the KorHF Registry, LVEF is associated with mortality, whereas LVEDD is only associated with readmission in a binary transformed form.

## Introduction

Acute heart failure (AHF) is one of the most common conditions encountered in emergency care facilities and hospitals and is associated with a poor prognosis worldwide [1–8]. Transthoracic echocardiography (TTE) is an important tool used for the initial evaluation of AHF and is recommended as the method of choice for screening AHF by both the European Society of Cardiology and the American Heart Association heart failure (HF) guidelines [9,10]. TTE provides useful information about morphological or functional changes and the etiology of AHF. Thus, in clinical practice, physicians often attempt to classify patients by echocardiographic parameters before estimating the prognoses. For example, patients with AHF may be classified as having mild, moderate or severe left ventricular (LV) dysfunction or dilatation based on LV ejection fraction (LVEF) or LV end-diastolic dimension (LVEDD) [11]. Although LVEF and LVEDD are representative echocardiographic parameters, their prognostic value is based on studies of patients with chronic HF, with little data from patients with AHF.

Therefore, we investigated whether the LVEF or LVEDD on echocardiogram can predict clinical outcomes in Korean patients with AHF.

## Materials and methods

### Criteria and enrollment

As described in a previous Korean Heart Failure (KorHF) Registry study [8], data describing the clinical status and outcomes of patients treated between June 2004 and April 2009 were obtained by reviewing registry data from 24 hospitals in Korea. The participating centers were evenly distributed across the nation proportional to the populations they serve. HF was diagnosed upon admission according to the Framingham criteria [12]. The Institutional Review Board or ethics committee at each participating hospital approved the study protocol (Institutional Review Board for Human Research at Konkuk University Hospital, KUH1010270), and the patients provided written informed consent prior to enrollment in the study.

### Data collection and follow-up

Patient demographic characteristics, baseline characteristics, underlying diseases, clinical presentations, treatments and outcomes from the initial presentation through hospitalization and at discharge were recorded. The data were entered into the KorHF Registry database via a web-based electronic data capture system that included an electronic case report form. The diagnosis of HF was required to be confirmed for each patient at the time of discharge. Guided by documented definitions, research coordinators used standardized report forms to collect the follow-up events until October 2009. When possible, medical records were reviewed when the patients required repeat hospitalization. In addition to patient telephone interviews, the referring physicians and institutions were contacted for additional information as necessary. We obtained information on patient survival and hospital readmission for HF. Events related to HF were defined as admission for HF deterioration, transplantation or cardiovascular death. The KorHF Registry Steering Committee of the Korean Society of Heart Failure performed the data collection and review.

### Etiology of HF

We attempted to determine the etiology of HF and to identify the demographic characteristics, clinical variables and biochemical markers with prognostic value. Ischemic HF was defined as a history of previous myocardial infarction (MI) or the presence of significant stenosis of more than 50% on a coronary angiogram.

### Echocardiography

An echocardiogram was performed after the patient was stable. LVEDD and LVEF were measured in M-mode images of the parasternal long axis according to the American Society of Echocardiography (ASE) guidelines. LVEF was calculated using Simpson’s method when a regional wall motion abnormality was observed. In our analysis, the cut-off values for LVEF and LVEDD were evaluated as quartile values due to the possibility of differences between Western and Korean patients.

### Clinical outcomes

We obtained information about patient survival and hospital readmission for HF. Events related to HF were defined as admission for HF deterioration, transplantation or cardiovascular death.

### Statistical analysis

Continuous variables are expressed as the mean ± standard deviation (SD). Groups were compared using one-way analysis of variance (ANOVA) or the χ2 test, as appropriate. Survival estimates were derived using the Kaplan-Meier method, and differences were tested using the log-rank test. After determining whether proportional hazards assumptions were violated, survival predictors were identified using multivariate Cox proportional hazards regression analyses. Backward selection was performed in each of the full data sets, with the criterion for inclusion set as p<0.10. The candidate independent variables with less than 10% missing values included age, gender, a previous diagnosis of HF, acute MI (AMI), diabetes mellitus (DM), chronic obstructive pulmonary disease (COPD), chronic kidney disease (CKD), atrial fibrillation (Af), systolic blood pressure (SBP), anemia, blood urea nitrogen (BUN) levels, serum sodium levels and LVEF or LVEDD at admission. All analyses were conducted using PASW software 17.0 (SPSS, Inc., an IBM Company, Chicago, IL, USA).

## Results

### Baseline characteristics of the study population

TTEs from 2,910 of the 3,200 patients in the KorHF Registry (90.9%) were evaluated. LVEF or LVEDD parameters were available for 2,887 patients. LVEF was measured in 73.4% of patients using M mode and in 34.6% by Simpson’s method (8% by both methods). The mean age of the 2,887 patients was 67.4±14.0 years old, and the proportion of male patients was 50.2%. Patients with a previous history of HF accounted for 28.5% of the total population, and 37.7% of the patients had HF with an ischemic etiology. The median LVEF was 37%, and approximately 73.4% of the patients exhibited NYHA III or NYHA IV disease at admission. Patients were classified into subgroups of LVEF (E1, E2, E3 and E4) or LVEDD (D1, D2, D3 and D4) by quartile values or by combined quartile values of LVEF and LVEDD. The baseline clinical, electrocardiographic and laboratory findings in each group and the medications used by patients in each group are summarized in Tables 1 and 2. The proportions of missing values for important variables are described in S1 Table. As the LVEF increased and LVEDD decreased, the patients tended to be older, have higher SBP at admission, have prior HF history and represent a greater proportion of non-ischemic HF cases.

**Table 1:** Baseline characteristics classified by LVEF quartiles.

	E1 (EF≥50%) (n = 653)	E2(37–49.9%) (n = 727)	E3(26–36.9%) (n = 708)	E4 (<25.9%) (n = 755)	p value
Age (yr)	70.2±13.3	68.9±13.9	67.6±13.7	63.4±5.5	<0.001
Male (%)	62.2	48.8	54.8	60.8	<0.001
BMI (kg/m^2^)	23.9±4.6	23.1±3.6	22.9±3.7	23.2±4.1	<0.001
SBP (mmHg)	136.3±30.1	133.2±31.1	128.8±28.8	136.3±30.1	<0.001
DBP (mmHg)	77.9±16.7	78.6±18.1	77.5±17.8	78.4±17.6	0.606
HR (beats/min)	85.3±26.0	91.1±26.0	92.6±23.9	93.9±23.0	<0.001
NYHA III, IV/I, II (%)	75.0	70.3	71.2	77.0	0.023
CHF (%)	27.9	25.0	26.3	34.8	<0.001
Ischemic etiology (%)	44.9	46.5	46.5	32.7	<0.001
CKD (%)	7.0	11.8	9.3	8.1	0.013
Stroke (%)	19.4	20.3	16.9	15.8	0.360
Hypertension (%)	52.7	50.9	47.7	35.4	<0.001
DM (%)	27.5	33.0	31.6	29.2	0.118
Af (%)	31.7	26.0	20.2	19.9	<0.001
Hematocrit	35.5	35.6	37.4	38.7	<0.001
Hb (g/dL)	12.0±2.3	12.0±2.3	12.6±2.4	13.1±2.3	<0.001
Cr (mg/dL)	1.37±1.07	1.60±1.55	1.50±1.4	1.41±0.96	0.002
BUN (mg/dL)	23.6±14.5	25.1±16.0	24.8±16.2	24.3±14.5	0.207
NT-proBNP (pg/mL)	5,547±7,742	8,878±10,472	9,914±10,485	9,076±9,257	<0.001
Serum sodium (mmol/L)	138.3±5.1	138.2±5.0	138.3±4.8	137.9±5.2	0.738
TC (mg/dL)	163.1±49.1	167.7±46.9	165.9±48.1	159.9±43.6	0.049
ACE inhibitor at OPD, n (%)	138 (24.0)	184 (30.3)	181 (30.0)	194 (31.0)	0.028
ARB, n (%)	124 (21.5)	150 (24.7)	156 (25.9)	146 (23.4)	0.345
Beta-blocker, n (%)	175 (30.3)	246 (40.5)	222 (36.7)	221 (33.8)	0.002
Diuretics, n (%)	255 (44.2)	275 (45.2)	294 (48.7)	312 (49.9)	0.144

BMI, body mass index; SBP, systolic blood pressure; DBP, diastolic blood pressure; HR, heart rate; NYHA, New York Heart Association functional class; CHF, congestive heart failure; CKD, chronic kidney disease; DM, diabetes mellitus; Af, atrial fibrillation; Hb, hemoglobin; BUN, blood urea nitrogen; Cr, serum creatinine; TC, total cholesterol; ACE, angiotensin-converting enzyme; and ARB, angiotensin receptor blocker.

P values were determined using chi-square tests or analyses of variance among the four groups.

**Table 2:** Baseline characteristics classified by LVEDD quartiles.

	D1 (EDD<50) (n = 753)	D2 (50–55.9) (n = 702)	D3 (56–62.9) (n = 615)	D4 (≥63) (n = 768)	p value
Age (yr)	71.2±13.4	69.4±13.6	66.7±13.7	62.6±5.2	0.076
Male (%)	35.1	43.3	56.7	65.5	<0.001
BMI (kg/m^2^)	22.8±4.4	23.2±3.6	23.4±3.8	23.6±4.0	0.012
SBP (mmHg)	132.4±31.1	132.8±29.9	132.4±28.4	126.5±27.2	0.015
DBP (mmHg)	77.7±17.3	78.2±17.1	79.3±17.8	77.6±18.2	0.133
HR (beats/min)	87.7±26.9	92.3±25.1	91.1±24.7	90.4±22.9	0.122
NYHA III, IV/I, II (%)	68.2	71.6	73.4	78.7	0.001
CHF (%)	20.6	22.7	31.8	39.1	<0.001
Hypertension (%)	51.0	49.1	47.9	37.6	<0.001
Ischemic etiology (%)	38.3	42.7	42.1	29.3	<0.001
DM (%)	30.1	34.5	34.4	23.4	<0.001
COPD (%)	3.5	3.2	4.3	2.6	0.441
CKD (%)	8.9	9.7	10.9	6.8	0.049
Stroke (%)	22.8	15.6	18.0	14.4	0.017
Atrial fibrillation (%)	26.3	23.4	25.5	22.8	0.403
Hematocrit	35.9±6.4	35.6±6.7	37.1±6.9	38.6±6.4	<0.001
Hemoglobin (g/dL)	12.2±2.6	12.1±2.4	12.5±2.4	13.0±2.6	<0.001
Cr (mg/dL)	1.42±1.28	1.53±1.42	1.51±1.27	1.43±1.07	0.243
BUN (mg/dL)	24.3±15.4	23.9±14.4	25.5±16.8	24.3±14.8	0.186
NT-proBNP (pg/dL)	6,811±8,759	8,966±10,304	9,637±10,635	8,278±9,015	<0.001
TC (mg/dL)	166.2±50.7	167.0±46.9	165.1±49.4	157.7±40.2	0.097
Serum sodium (mmol/L)	138.1±5.2	138.1±4.9	138.3±5.3	138.1±4.9	0.753
ACE, n (%)	163 (25.4)	175 (29.2)	163 (31.0)	203 (31.4)	0.076
ARB, n (%)	148 (23.1)	138 (23.0)	118 (22.4)	169 (26.1)	0.417
Beta-blocker, n (%)	235 (36.6)	222 (37.0)	182 (34.6)	215 (33.0)	0.465
Diuretics, n (%)	296 (36.6)	250 (41.7)	255 (48.5)	339 (52.4)	0.002

### Clinical outcomes that depend on LVEF or LVEDD

**Fig 1:**
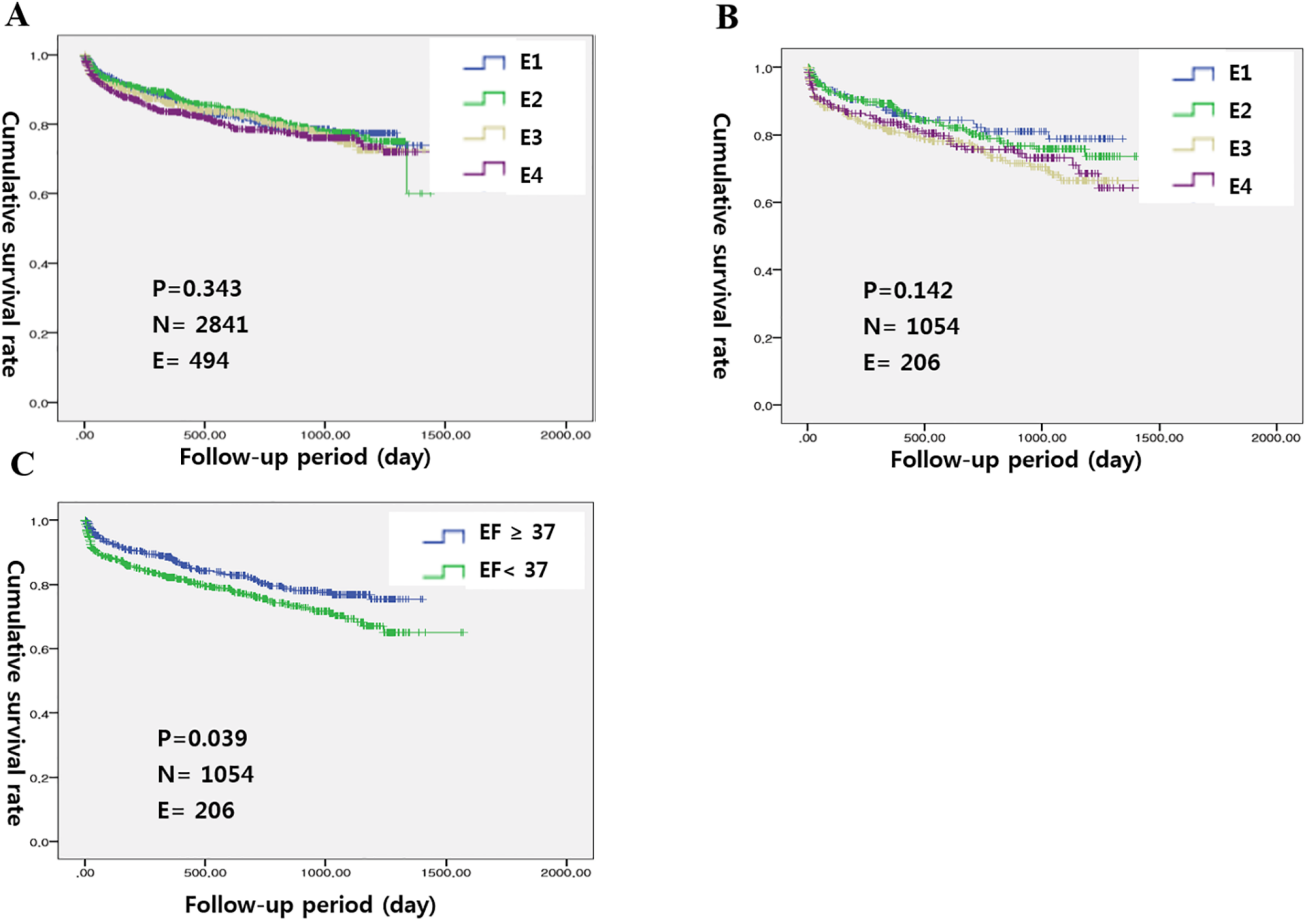
Unadjusted Kaplan-Meier free from cardiovascular mortality survival curves for (A) all patients, (B) patients with ischemic HF based on LVEF (quartiles) and (C) patients with ischemic HF based on LVEF (median).

During the median follow-up period of 17.2 months (IQR 5.5, 32.2), 499 (17.3%) cardiovascular deaths and 767 (26.6%) episodes with readmission occurred among the 2,884 patients. No significant differences in the median follow-up duration were observed among the four groups based on either LVEF or LVEDD (p = 0.078 and 0.174, respectively). Unadjusted survival curves generated based on quartiles of either LVEF or LVEDD did not differ significantly among patients (p = 0.343 and 0.142, respectively); however, in patients with ischemic HF, an LVEF greater than the median value correlated with a better prognosis (log-rank test; p = 0.039) (Fig 1).

According to the results of the multivariate Cox proportional hazards model, age, LVEF (dichotomized), a history of HF, SBP at admission, serum sodium levels, hemoglobin and BUN levels are associated with cardiovascular mortality in patients with ischemic HF (S2 Table). After SBP, serum sodium, Hb, BUN and age were binary-transformed, LVEF was a significant predictor of cardiovascular mortality only in patients with ischemic HF using a Cox model that included a history of HF, age, SBP, serum sodium levels, sex, DM, COPD, CKD, AMI, Af and anemia (hazard ratio (HR) 1.475, 95% confidence interval (CI) 1.099–1.979, p = 0.010) (Table 3). LVEF was also a significant predictor of cardiovascular mortality in patients with both ischemic HF and reduced LVEF (HR 1.413, 95% CI 1.023–1.952, p = 0.036).

**Table 3:** Multivariate cox proportional hazards model for death in patients with ischemic HF.

Endpoint		Death		
		HR	95% CI	p value
EF *		1.434	1.056–1.946	0.021
	+ Hx of CHF	1.413	1.040–1.819	0.027
	+ Age	1.435	1.057–1.949	0.021
	+ SBP	1.386	1.016–1.891	0.039
	+ Serum sodium	1.386	1.013–1.896	0.041
	+ BUN	1.367	0.999–1.870	0.051
	+ NYHA(III+IV)	1.301	0.937–1.807	0.116

*Hazard ratio of LVEF <median (37%) versus ≥37% adjusted by sex, DM, COPD, CKD, AMI, Af and Hb.

Cut-off values for binary conversion: age, 70; sodium, 135; SBP, 110; Hb, 12; and BUN, 32.

**Fig 2:**
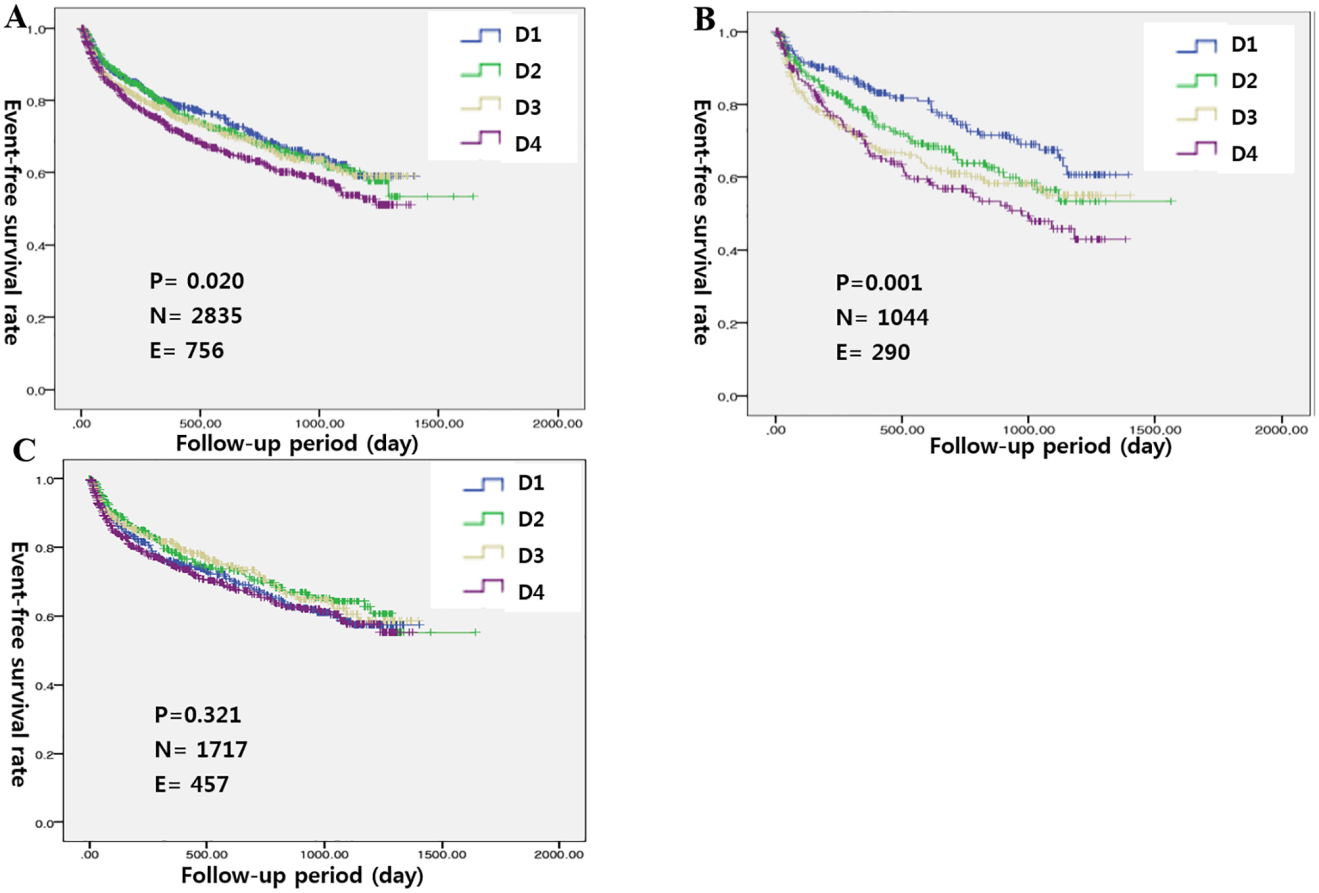
Readmission-free survival curves based on LVEDD (quartiles) for (A) all patients, (B) patients with ischemic HF and (C) patients with non-ischemic HF.

**Fig 3:**
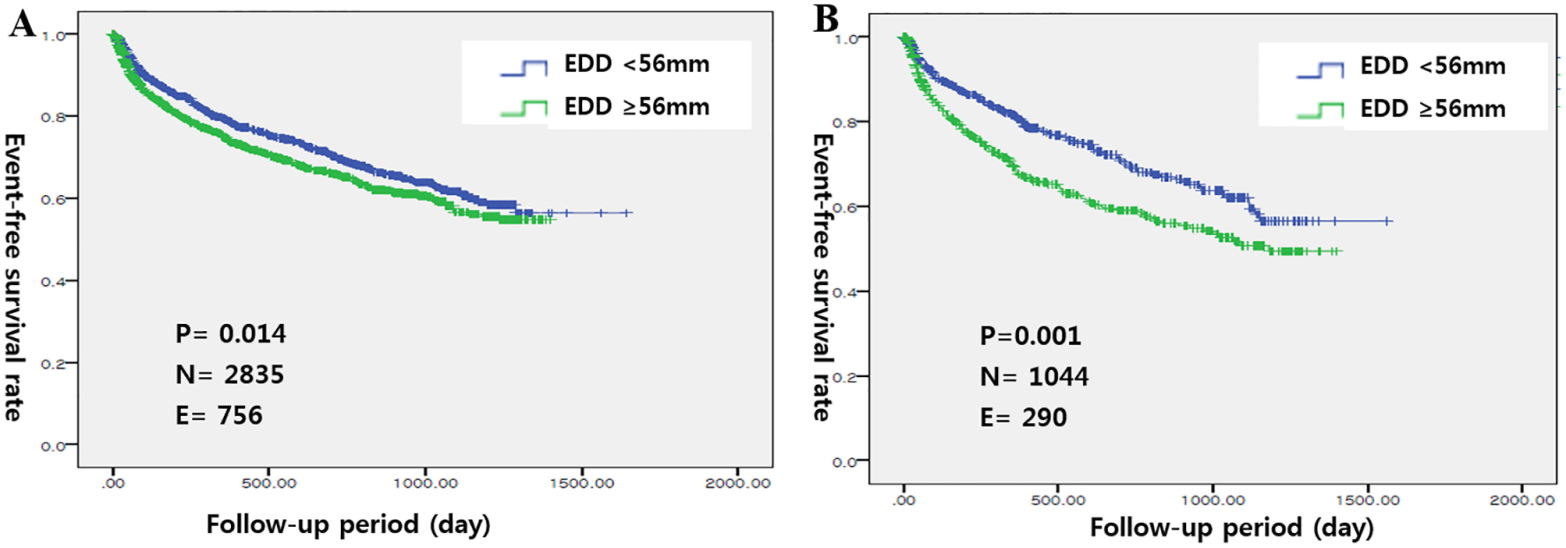
Readmission-free survival curves based on LVEDD (quartiles) in A) overall patients and B) ischemic HF patients.

**Fig 4:**
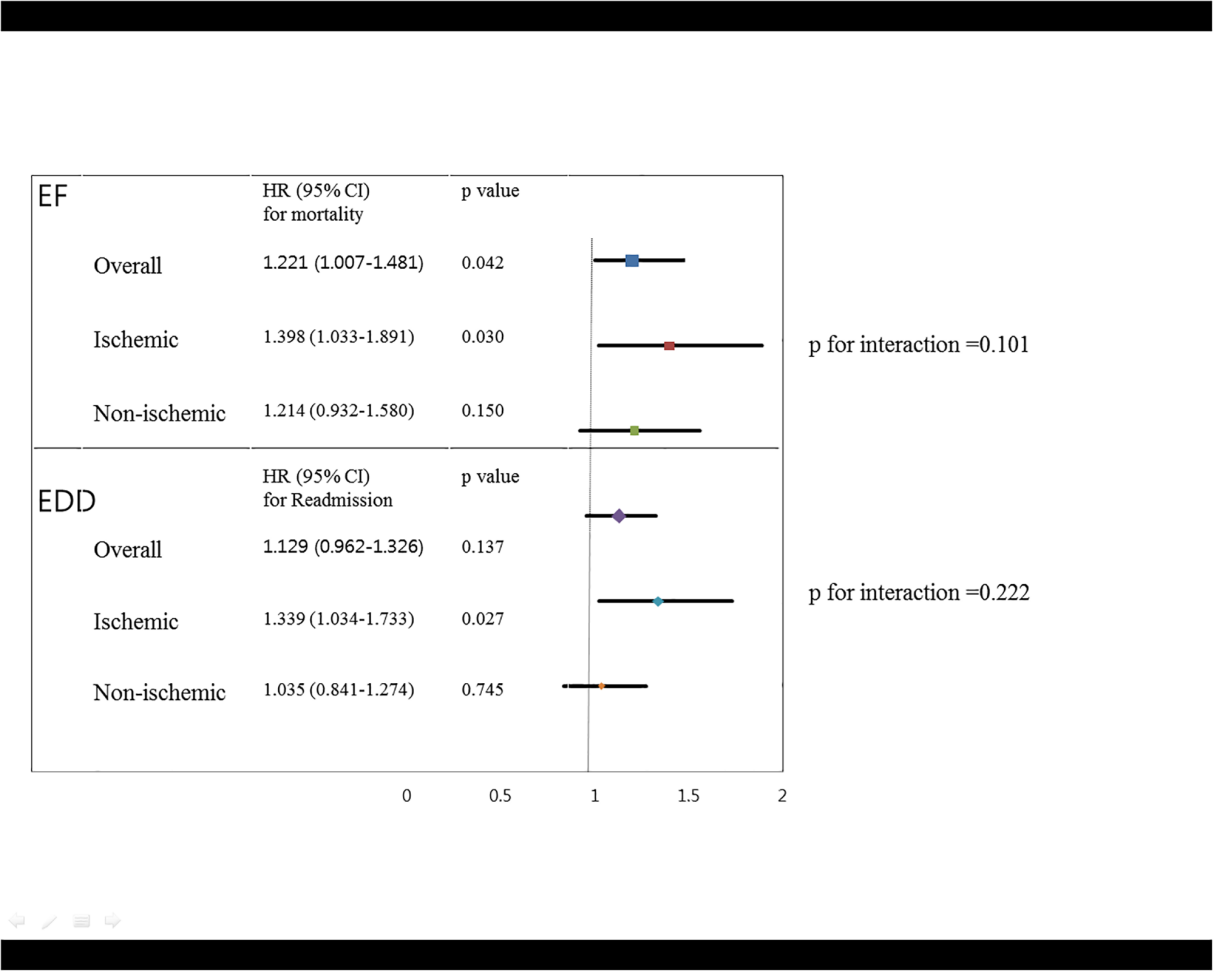
Ability of LVEF or LVEDD to predict cardiovascular outcome.

Cumulative readmission-free survival rates differed significantly among patients classified based on either quartiles or dichotomous LVEDD, and significant differences were observed among patients with ischemic HF (Figs 2 and 3). In the multivariate Cox proportional hazards regression analyses, LVEDD remained significant only in patients with ischemic HF after adjusting for sex, age, AMI, DM, COPD, serum sodium levels, SBP, BUN levels and anemia (HR 1.401, 95% CI 1.067–1.841, p = 0.015) (Table 4) (Fig 4).

**Table 4:** Multivariate cox proportional hazards model for readmission of patients with ischemic HF.

Endpoint		Readmission		
		HR	95% CI	p value
LVEDD *		1.396	1.079–1.805	0.011
	+DM	1.398	1.064–1.836	0.016
	+HTN	1.401	1.067–1.841	0.015
	+Hx of CHF	1.286	0.972–1.701	0.078

*Hazard ratio of LVEDD ≥median (56 mm) versus <56 mm adjusted for sex, AMI, COPD, serum sodium levels, SBP, BUN levels, age and Hb.

Unadjusted survival curves based on either LVEF or LVEDD did not significantly predict death or readmission among patients with dilated cardiomyopathy (DCMP; n = 536) (data not shown).

## Discussion

This study investigated the role of echocardiographic parameters in predicting the clinical outcomes of Korean patients with AHF and identified clinical differences among patients based on subgroups of echocardiographic parameters. In this study, two representative echocardiographic parameters, LVEF and LVEDD, were found to be associated with the clinical outcomes of patients with ischemic HF in a large, multicenter, real-world AHF registry. Interestingly, LVEF was closely associated with only cardiovascular mortality and not with the readmission rate of patients with ischemic HF, whereas LVEDD was only significantly associated with readmission.

Although data on the association between echocardiographic parameters and the clinical outcomes of patients with AHF are lacking, some information is available from studies of patients with chronic HF and randomized clinical trials.

According to the results of the DIG trial (n = 7,788; mean age, 63 yr), in which most of the patients (70%) had HF with an ischemic etiology [13], an LVEF of less than 40% had an incremental prognostic value for mortality in patients with chronic HF. In the Ontario-EFFECT study [14], LV dysfunction of less than 40% was associated with an age-adjusted HR of 1.55. In these studies, although LVEF was measured using methods such as angiography and the nuclear method rather than by echocardiography, the results are consistent with the results of our study.

In a subgroup (n = 336) of the BEST trial [15], which included 2,708 patients with advanced HF, NYHA III/IV and an LVEF <35%, the LV end-diastolic volume index was associated with death and readmission in the multivariate analysis. However, LVEDD was significant in only the univariate analysis, suggesting that LVEDD volume may have better prognostic value than LVEDD dimension (the only parameter available in our study). In the Val-HeFT study (n = 5,010; 63 yr; mean LVEF: 27%) [16], both baseline LVEDD and LVEF were powerful predictors of mortality or mortality and morbidity combined, suggesting that both LVEDD and LVEF reflect the severity of the remodeling process.

In contrast to the studies described above, LV hypertrophy on echocardiogram showed a better association with prognosis than LVEF or LVEDD in the SOLVD study [17], which was partially explained by the enrollment of a greater number of patients with hypertension and preserved HF. Accordingly, LVEF or LVEDD tends to predict clinical outcomes in HF patients with an ischemic etiology or in patients with a more advanced stage of HF. This finding may explain why we failed to observe an association between either LVEF or LVEDD and clinical outcomes in all patients with HF in the present study, in which only 38% of patients had ischemic HF.

Unlike previous studies, this study failed to identify a prognostic role for LVEF or LVEDD in patients with AHF complicated with DCMP. One possible explanation is that some patients with severe LV dysfunction and a reversible underlying cause, such as hypertensive, tachycardia-induced or alcoholic HF, may be initially diagnosed with DCMP because of the very similar clinical presentation of AHF at the time of enrollment.

Although the question remains as to why the echocardiographic parameters are only predictive for ischemic AHF, the finding that echocardiographic parameters such as the LVEF and LVEDD predict outcomes in patients with ischemic AHF, similar to patients with chronic HF, is useful.

This study raises several issues. First, we may need to use other echocardiographic parameters to predict clinical outcomes in patients with non-ischemic AHF due to differences in LV remodeling patterns depending on the etiology of HF, as noted in the SOLVD study. Second, the heterogeneity of patients with HF may have affected the analysis. In general, the non-ischemic HF group was more heterogeneous than the ischemic HF group due to differences in etiology. However, even within one etiological type, such as DCMP, a wide spectrum of prognoses has been reported due to the complex composition of patients [18]. Thus, not surprisingly, neither the LVEF nor LVEDD was significantly associated with clinical outcomes in our study.

Third, LVEF and LVEDD each reflect different conditions in patients with AHF. During clinical follow-up, LV systolic function may decrease following an acute change in stress, thereby increasing the risk of death. Meanwhile, the fundamental LV size does not rapidly change; rather, it dilates gradually. Thus, LVEDD may reflect the chronicity of disease, which may result in frequent readmission.

### Study limitations

Several limitations of this study must be acknowledged. First, we did not reanalyze the raw LVEF/LVEDD data in the central lab, and the timing of the echocardiograms varied according to the patient’s condition and the institution’s policy, which may influence the LVEF and LVEDD measurements. Because the measurement techniques were similar across the LVEF/LVEDD quartile groups, any misclassification of LVEF or LVEDD would presumably have been evenly distributed and, thus, should produce no net bias. Next, we could not evaluate the effect of changes in LVEF and LVEDD on medication use and the prognosis of patients with acute HF. Finally, the subgroup sample sizes, the duration of the follow-up period and the number of covariables are limited, suggesting the potential presence of residual confounders despite our efforts at adjustment. Therefore, additional large-scale studies might be needed to better characterize the association between LVEF/LVEDD and clinical outcomes in acute HF patients with specific etiologies.

## Conclusions

Among patients with ischemic AHF in the KorHF Registry, LVEF is associated only with cardiovascular mortality and not with readmission, whereas LVEDD is associated only with readmission only as a binary transformed form.

## Supporting information

S1 Table Candidate predictors of clinical outcomes. (DOCX)

S2 Table Univariate and multivariate cox proportional hazards models of death in patients with ischemic HF. (DOCX)
