# Supplementary material for: The limited prognostic role of echocardiograms in short-term follow-up after acute decompensated heart failure: An analysis of the Korean Heart Failure (KorHF) Registry
Source: PLoS One. 2017 Dec 19;12(12):e0188938. doi: 10.1371/journal.pone.0188938 (PMC5736190; doi:10.1371/journal.pone.0188938)
Supplement: S1 Table — (DOCX) [file pone.0188938.s001.docx]

**S1 Table. Candidate Predictors of Clinical Outcomes.**

| Variable | N | n | Distribution (%) of dichotomous  25th percentile, median and 75th percentile values for continuous variables | Missing  % |
| --- | --- | --- | --- | --- |
| Age (yr) | 2,887 |  | 60, 70, 78 | 0 |
| SBP (mmHg) | 2,824 |  | 110, 130, 149 | 2.9 |
| Hb (g/dl) | 2,879 |  | 10.9, 12.5, 14.1 | 0.3 |
| BUN (mg/dl) | 2,860 |  | 15.0, 19.9, 29 | 0.9 |
| Serum sodium (mmol/l) | 2,610 |  | 136, 139, 141 | 9.6 |
| NYHA (III/IV) | 2,456 | 1,803 | 73.4 | 14.9 |
| Sex (male) | 2,887 | 1,448 | 50.2 | 0 |
| Hx of CHF | 2,650 | 755 | 28.5 | 8.2 |
| Hx of AMI | 2,886 | 397 | 13.8 | 0.0 |
| Hx of HTN | 2,886 | 1,337 | 46.3 | 0.0 |
| Hx of DM | 2,886 | 877 | 30.4 | 0.0 |
| Hx of COPD | 2,650 | 91 | 3.4 | 8.2 |
| Hx of Af | 2,641 | 645 | 24.4 | 8.5 |
| Hx of CKD | 2,641 |  | 9.0 | 8.5 |
