# Supplementary material for: The limited prognostic role of echocardiograms in short-term follow-up after acute decompensated heart failure: An analysis of the Korean Heart Failure (KorHF) Registry
Source: PLoS One. 2017 Dec 19;12(12):e0188938. doi: 10.1371/journal.pone.0188938 (PMC5736190; doi:10.1371/journal.pone.0188938)
Supplement: S2 Table — (DOCX) [file pone.0188938.s002.docx]

**S2 Table. Univariate and Multivariate Cox Proportional Hazards Models of Death in Patients with Ischemic HF**.

| Variable | Univariate | | | Multivariate | | |
| --- | --- | --- | --- | --- | --- | --- |
|  | HR | 95% CI | p value | HR | 95% CI | p value |
| Sex | 1.015 | 0.733-1.408 | 0.927 |  |  |  |
| Hx of CHF | 1.373 | 0.966-1.408 | 0.077 | 1.387 | 1.270-2.155 | 0.040 |
| Hx of DM | 1.116 | 0.793-1.570 | 0.528 |  |  |  |
| COPD | 1.533 | 0.779-2.943 | 0.199 |  |  |  |
| CKD | 1.294 | 0.815-2.055 | 0.274 |  |  |  |
| SBP* | 0.989 | 0.985-0.994 | <0.001 | 0.992 | 0.987-0.997 | 0.002 |
| BUN* | 1.015 | 1.009-1.020 | <0.001 | 1.012 | 1.005-1.020 | 0.001 |
| LVEF | 1.367 | 0.999-1.870 | 0.051 | 1.475 | 1.099-1.979 | 0.010 |
| Hx of AMI | 1.112 | 0.785-1.575 | 0.550 |  |  |  |
| Af | 1.021 | 0.689-1.513 | 0.918 |  |  |  |
| Serum sodium* | 0.952 | 0.933-0.971 | <0.001 | 0.964 | 0.940-0.988 | 0.003 |
| Age* | 1.029 | 1.017-1.041 | <0.001 | 1.023 | 1.008-1.038 | 0.002 |
| Hb* | 0.863 | 0.820-0.909 | <0.001 | 0.964 | 0.940-0.988 | 0.001 |

* Continuous variable
